# Linagliptin, a Selective DPP-4 Inhibitor, Attenuates Ketamine- and Diazepam-Induced Deficits in Passive Avoidance Performance in Mice

**DOI:** 10.3390/brainsci16070710

**Published:** 2026-06-30

**Authors:** Krzysztof Fronc, Piotr Listos, Paulina Kasprzak, Marcin Berger, Tymoteusz Słowik, Jolanta Kotlińska, Ewa Poleszak, Irena Baranowska-Bosiacka, Listos Emilia, Małgorzata Łupina, Adrian Pysiewicz, Joanna Listos

**Affiliations:** 1 Department of Pharmacology and Pharmacodynamics, Medical University of Lublin, Chodźki 4a St., 20-093 Lublin, Poland; krzysztof.michal.fronc@gmail.com (K.F.); jolanta.kotlinska@umlub.edu.pl (J.K.); listosemilia@gmail.com (L.E.); adrian.pysiewicz@gmail.com (A.P.); 2 Department of Pathomorphology and Forensic Medicine, Faculty of Veterinary Medicine, University of Life Sciences, Głęboka 30 St., 20-612 Lublin, Poland; 3 Department of Conservative Dentistry with Endodontics, Medical University of Lublin, Chodźki 6 St., 20-093 Lublin, Poland; paulina.kasprzak@umlub.edu.pl; 4 Department of Oral Surgery, Medical University of Lublin, Chodźki 6 St., 20-093 Lublin, Poland; marcin.berger@umlub.edu.pl; 5 Experimental Medicine Center, Medical University of Lublin, Jaczewskiego 8d St., 20-090 Lublin, Poland; tymoteusz.slowik@umlub.edu.pl; 6 Department of Applied and Social Pharmacy, Medical University of Lublin, Chodźki 1 St., 20-093 Lublin, Poland; ewa.poleszak@umlub.edu.pl; 7 Department of Biochemistry and Medical Chemistry, Pomeranian Medical University, Powstańców Wlkp. 72 Av., 70-111 Szczecin, Poland; irena.baranowska.bosiacka@pum.edu.pl; 8 Department of Experimental and Clinical Pharmacology, Medical University of Lublin, Jaczewskiego 8b St., 20-090 Lublin, Poland; malgorzata.lupina@umlub.edu.pl

**Keywords:** GLP-1 peptide, diazepam, ketamine, memory impairments, ELISA, BDNF, passive avoidance test

## Abstract

**Highlights:**

- Linagliptin attenuated ketamine-induced passive avoidance deficits in mice.
- Linagliptin increased BDNF levels in the prefrontal cortex but not in the hippocampus.

**Abstract:**

Background: Linagliptin, a potent and highly selective dipeptidyl peptidase-4 (DPP-4) inhibitor approved for the treatment of type 2 diabetes, enhances glucagon-like peptide-1 (GLP-1) signaling. Because GLP-1 receptors are widely expressed in the brain, DPP-4 inhibitors have emerged as potential modulators of central nervous system function. The present study investigated the effects of linagliptin (10 and 20 mg/kg, i.p.) on ketamine- (10 mg/kg, i.p.) and diazepam-induced (2 mg/kg, i.p.) deficits in passive avoidance performance in mice. Behavioral effects were assessed using the passive avoidance test, and brain-derived neurotrophic factor (BDNF) levels in the prefrontal cortex and hippocampus were determined by enzyme-linked immunosorbent assay (ELISA). Linagliptin attenuated ketamine- and diazepam-induced deficits in passive avoidance performance. In addition, both acute and chronic administration of linagliptin increased BDNF levels in the prefrontal cortex but not in the hippocampus. These findings provide preliminary evidence that linagliptin modulates passive avoidance performance in mice and is associated with increased BDNF levels in the prefrontal cortex. Further studies employing complementary behavioral paradigms and additional molecular approaches are required to clarify the neuropharmacological mechanisms underlying these effects.

## 1. Introduction

Linagliptin is a dipeptidyl peptidase-4 (DPP-4) inhibitor approved for the treatment of type 2 diabetes. By inhibiting DPP-4, linagliptin prevents the degradation of incretin hormones, thereby prolonging their biological activity and improving glycemic control [1]. Unlike other DPP-4 inhibitors, linagliptin exhibits a unique pharmacokinetic profile characterized by concentration-dependent protein binding and minimal renal excretion, eliminating the need for dose adjustment according to age, body weight, or renal function [2,3].

Pharmacodynamically, linagliptin is a potent and highly selective DPP-4 inhibitor that produces a dose-dependent increase in circulating glucagon-like peptide-1 (GLP-1) levels [3]. Although GLP-1 is primarily recognized for its role in glucose homeostasis, its receptors are widely expressed throughout the central nervous system, including the hippocampus, prefrontal cortex, hypothalamic nuclei, and brainstem [4,5,6,7,8,9]. This widespread distribution enables GLP-1 to act as a neuromodulator involved in multiple neuronal processes [10,11]. Experimental evidence indicates that GLP-1 modulates both N-methyl-D-aspartate (NMDA)- and γ-aminobutyric acid (GABA)-mediated neurotransmission [12,13] while also reducing neuroinflammation and oxidative stress, promoting neuronal survival, and supporting learning and memory [14]. Consequently, GLP-1 receptor agonists have attracted considerable interest as potential therapeutic agents for several neurological disorders, including Alzheimer’s disease [15], Parkinson’s disease [16], ischemic stroke [17], and multiple sclerosis [18].

The neuroprotective effects of GLP-1 are thought to be mediated, at least in part, through regulation of brain-derived neurotrophic factor (BDNF), a key mediator of neuronal survival and synaptic plasticity [19,20]. GLP-1 has been shown to enhance hippocampal BDNF expression through activation of the cAMP/PKA/CREB signaling pathway [21,22,23], thereby linking metabolic signaling with neuronal plasticity and cognitive function [24,25]. Consistent with these findings, several studies suggest that DPP-4 inhibitors exert neuroprotective effects by reducing oxidative stress, attenuating neuroinflammation, and improving synaptic plasticity [26,27]. Linagliptin has also been reported to improve cognitive performance in a rat model of Alzheimer’s disease [28], whereas another DPP-4 inhibitor, sitagliptin, attenuated dopaminergic neurodegeneration in an experimental model of Parkinson’s disease [29]. Nevertheless, knowledge of the neuropharmacological actions of DPP-4 inhibitors remains limited. In our previous study, linagliptin enhanced serotonergic, noradrenergic, and dopaminergic neurotransmission in the striatum and hippocampus, supporting its neuromodulatory properties [30]. However, its effects in other experimental models and its influence on BDNF expression have not yet been investigated.

Therefore, the present study examined the effects of linagliptin on ketamine- and diazepam-induced deficits in passive avoidance performance in mice. Ketamine and diazepam impair performance in the passive avoidance test through distinct pharmacological mechanisms involving NMDA receptor antagonism and GABAergic modulation, respectively [31]. Increasing evidence suggests that DPP-4 inhibitors may influence cognitive function through GLP-1-dependent mechanisms and the regulation of neurotrophic factors [32]; however, whether linagliptin can attenuate behavioral deficits induced by acute NMDA receptor blockade or enhanced GABAergic neurotransmission remains unclear. To address this question, the passive avoidance test was used to evaluate the effects of acute and chronic linagliptin administration on the acquisition and consolidation of an aversive task, as well as following repeated treatment. In addition, because BDNF is a critical regulator of synaptic plasticity, its levels were determined in the prefrontal cortex and hippocampus using an enzyme-linked immunosorbent assay (ELISA). By combining behavioral and molecular approaches, this study aimed to further characterize the neuropharmacological profile of linagliptin and to provide preliminary evidence regarding its effects on passive avoidance performance and BDNF expression in selected brain regions.

## 2. Materials and Methods

### 2.1. Animals

The experiments were performed on male Swiss albino mice weighing 20–25 g. Animals were housed in groups of 4–5 per cage under controlled environmental conditions (22 ± 1 °C) and maintained on a standard 12 h light/dark cycle. Standard laboratory chow (Murigran, Motycz, Poland) and water were available ad libitum. The cages were provided with environmental enrichment. Before the experiments, the animals were allowed to acclimate to the laboratory conditions for 7 days. All behavioral experiments were conducted in a well-lit, soundproofed room. A total of 360 mice were used in the study.

All experimental procedures were conducted in accordance with the National Institutes of Health Guide for the Care and Use of Laboratory Animals and the European Community Council Directive for the Care and Use of Laboratory Animals. The study protocol was approved by the Local Ethics Committee for Animal Experiments at the Medical University of Lublin (approval no. 36/2023).

### 2.2. Drugs

Linagliptin (MedChemExpress, Monmouth Junction, NJ, USA) was administered at doses of 10 and 20 mg/kg to evaluate the effects of DPP-4 inhibition in mice. Memory deficits were induced using ketamine (10 mg/kg; Pfizer, New York, NY, USA) or diazepam (2 mg/kg; Pfizer, New York, NY, USA). The doses of ketamine and diazepam were selected based on previous studies conducted in our Department, demonstrating their ability to induce memory impairment without producing confounding sedative or motor effects. This was further supported by the comparable exploratory behavior and baseline transition latencies (TL1) observed across all experimental groups. All compounds were administered intraperitoneally (i.p.) at a volume of 0.1 mL/10 g body weight.

Linagliptin was initially dissolved in 3–4 drops of 96% ethanol (final ethanol concentration < 0.1%) and subsequently diluted with physiological saline to the required volume. Diazepam and ketamine were dissolved in 0.9% NaCl solution. No behavioral differences were observed between animals receiving saline alone and those receiving saline containing ethanol. Control animals (vehicle group) received an equivalent volume of the appropriate vehicle.

For each behavioral experiment, the animals were randomly assigned to six experimental groups: vehicle, ketamine, ketamine + linagliptin (10 mg/kg), ketamine + linagliptin (20 mg/kg), linagliptin (10 mg/kg), and linagliptin (20 mg/kg). The experimental design for the diazepam studies was identical, except that ketamine was replaced with diazepam.

### 2.3. Apparatus

Passive avoidance performance was assessed using the passive avoidance test, a well-established behavioral paradigm for evaluating retention of an aversive experience in rodents based on their innate preference for dark environments. The apparatus (Bio-Sys, Wrocław, Poland) consisted of two compartments (10 × 13 × 15 cm) separated by a guillotine door: an illuminated compartment equipped with an 8 W fluorescent lamp and a dark, non-illuminated compartment. The floor of both compartments consisted of stainless-steel grid rods connected to a shock generator capable of delivering a mild electric foot shock.

The passive avoidance test was performed over two consecutive days. On the first experimental day, each mouse was placed individually into the illuminated compartment and allowed to habituate for 30 s. The guillotine door was then opened, allowing free access to the dark compartment. Once the animal had completely entered the dark compartment (all four paws inside), the guillotine door was closed and a mild electric foot shock (0.2 mA, 2 s) was delivered through the grid floor. This stimulus intensity is considered relatively low for mice. The animal was then immediately returned to its home cage.

The time elapsed between opening the guillotine door and complete entry into the dark compartment was recorded as transition latency 1 (TL1). Animals that did not enter the dark compartment within 300 s were gently placed into the dark compartment, after which the foot shock was delivered. In these cases, TL1 was assigned a value of 300 s.

Twenty-four hours later, the retention session was performed under identical conditions, except that no foot shock was delivered. Transition latency 2 (TL2) was recorded. If the animal did not enter the dark compartment within 300 s, the session was terminated and TL2 was recorded as 300 s.

Passive avoidance performance was quantified using the SCORE calculated as:

SCORE = (TL2 − TL1)/TL1

This index normalizes retention latency to the individual baseline latency and provides a relative measure of behavioral performance. Higher SCORE values indicate better retention of the aversive experience, whereas lower values indicate impaired passive avoidance performance. SCORE values were compared across all experimental groups.

### 2.4. Behavioral Assay

#### 2.4.1. Effects of Acute and Chronic Linagliptin Administration on Ketamine-Induced Passive Avoidance Deficits in Mice-Experimental Design

The effects of linagliptin on ketamine-induced deficits in passive avoidance performance were evaluated using three experimental paradigms: acquisition, consolidation, and chronic treatment.

In the acquisition paradigm, linagliptin was administered during the acquisition phase of ketamine-induced behavioral deficits. On the first experimental day, ketamine (10 mg/kg) was administered, followed 5 min later by linagliptin (10 or 20 mg/kg). Forty-five minutes after linagliptin administration, the passive avoidance training session was performed and TL1 was recorded. Twenty-four hours later, the retention session was conducted and TL2 was measured. No injections or aversive stimuli were administered on the second day.

In the consolidation paradigm, linagliptin was administered immediately after the training session to assess its effects on memory consolidation. Fifty minutes after ketamine administration, TL1 was recorded, after which mice received linagliptin (10 or 20 mg/kg). Twenty-four hours later, TL2 was measured under the same conditions as in the acquisition paradigm, without additional drug administration or foot shock.

**Figure 1:**
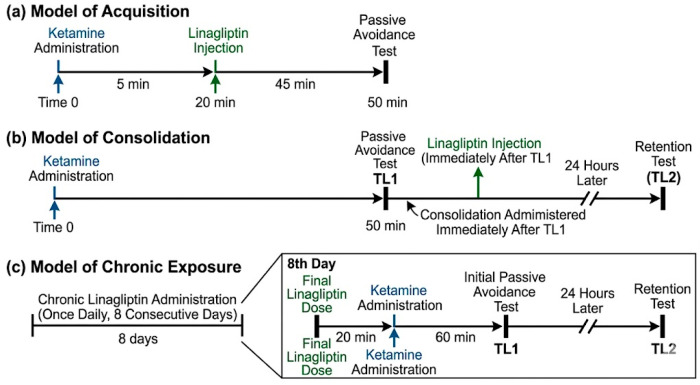
Graphical presentation of experimental protocol with ketamine.

In the chronic treatment paradigm, linagliptin (10 or 20 mg/kg, i.p.) was administered once daily for eight consecutive days. On day 8, ketamine (10 mg/kg, i.p.) was injected 20 min after the final linagliptin dose. Fifty minutes later, the training session was performed and TL1 was recorded. The retention session (TL2) was conducted 24 h later without further drug administration or aversive stimulation. Graphical presentation of experimental protocol with ketamine is shown in Figure 1.

#### 2.4.2. Effects of Acute and Chronic Linagliptin Administration on Diazepam-Induced Passive Avoidance Deficits in Mice—Experimental Design

As in the ketamine model, the effects of linagliptin were evaluated using three experimental paradigms: acquisition, consolidation, and chronic treatment.

In the acquisition paradigm, diazepam (2 mg/kg) was administered first, followed 20 min later by linagliptin (10 or 20 mg/kg). Forty-five minutes after linagliptin administration, the passive avoidance training session was performed and TL1 was recorded. Twenty-four hours later, the retention session was conducted and TL2 was measured. No injections or aversive stimuli were administered on the second day.

In the consolidation paradigm, diazepam (2 mg/kg) was administered, and TL1 was recorded 65 min later. Immediately after the training session, mice received linagliptin (10 or 20 mg/kg). Twenty-four hours later, TL2 was measured under the same conditions, without additional drug administration or foot shock.

**Figure 2:**
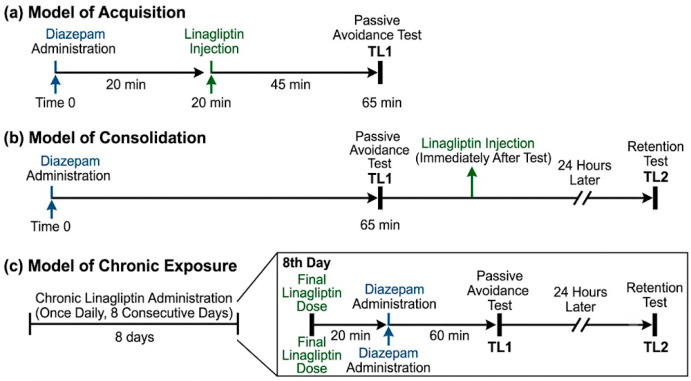
Graphical presentation of experimental protocol with diazepam.

In the chronic treatment paradigm, linagliptin (10 or 20 mg/kg, i.p.) was administered once daily for eight consecutive days. On day 8, diazepam (2 mg/kg, i.p.) was injected 20 min after the final linagliptin dose. Fifty minutes later, the training session was performed and TL1 was recorded. The retention session (TL2) was conducted 24 h later without further drug administration or aversive stimulation. Graphical presentation of experimental protocol with ketamine is shown in Figure 2.

Following the behavioral experiments, the mice were euthanized by decapitation. The prefrontal cortex and hippocampus were rapidly dissected, immediately frozen, and stored at −80 °C until further analysis.

### 2.5. Tissue Homogenization

Prior to the immunoenzymatic analyses, tissue samples were homogenized. Briefly, each sample was weighed and transferred to a round-bottom tube. All procedures were performed at a temperature not exceeding 4 °C. Lysis buffer (Cloud-Clone Corp., Wuhan, China; Cat. No. IS007) supplemented with protease inhibitors (Thermo Fisher Scientific, Waltham, MA, USA; Cat. No. 87786) was added to each sample. Homogenization was performed immediately using an Ultra-Turrax homogenizer (IKA, T10 basic ULTRA-TURRAX, Staufen, Germany) for approximately 45–60 s. The homogenates were then centrifuged at 11,000 rpm for 20 min at 4 °C. Following centrifugation, the supernatants were collected and stored at −80 °C until further analysis.

### 2.6. Determination of Total Protein and BDNF Levels

The total protein concentration in each brain sample was determined using a bicinchoninic acid (BCA) protein assay (BCA Protein Quantification Kit, Cloud-Clone Corp., Katy, TX, USA; Cat. No. IS003).

BDNF levels were determined using a commercially available sandwich enzyme-linked immunosorbent assay (ELISA) kit (Cloud-Clone Corp., Katy, TX, USA; Cat. No. SEA011Mu) according to the manufacturer’s instructions. Briefly, a standard curve was generated using serial dilutions of the BDNF standard. Samples and standards were added to microplate wells pre-coated with a specific anti-BDNF antibody, followed by incubation with a biotin-conjugated anti-BDNF antibody and horseradish peroxidase-conjugated avidin. Subsequently, 3,3′,5,5′-tetramethylbenzidine (TMB) substrate was added, resulting in color development proportional to the amount of bound BDNF. The reaction was terminated by the addition of sulfuric acid, and absorbance was measured spectrophotometrically at 450 nm.

### 2.7. Statistical Analysis

Statistical analyses were performed using GraphPad Prism version 8.3.0 (GraphPad Prism Software, version 8.3.0, GraphPad Software, San Diego, CA, USA).

Behavioral data were analyzed using one-way analysis of variance (ANOVA) followed by Tukey’s post hoc test for multiple comparisons. Data are presented as mean SCORE ± standard error of the mean (SEM). Each experimental group included 8–10 animals (NB = 8–10).

The normality of the immunoenzymatic data was assessed using the Shapiro–Wilk test. As the data were not normally distributed, comparisons between groups were performed using the non-parametric Mann–Whitney U test. Calculations of total protein concentration and BDNF levels were performed using Microsoft Excel 2021. The immunoenzymatic results are presented as mean BDNF concentration (ng) ± standard deviation (SD) normalized to total protein content (μg) in the prefrontal cortex or hippocampus. Each experimental group included 5–6 samples (NI = 5–6).

For all analyses, a value of *p* < 0.05 was considered statistically significant.

## 3. Results

### 3.1. Effects of Acute and Chronic Linagliptin Administration on Ketamine-Induced Passive Avoidance Deficits in Mice

**Figure 3:**
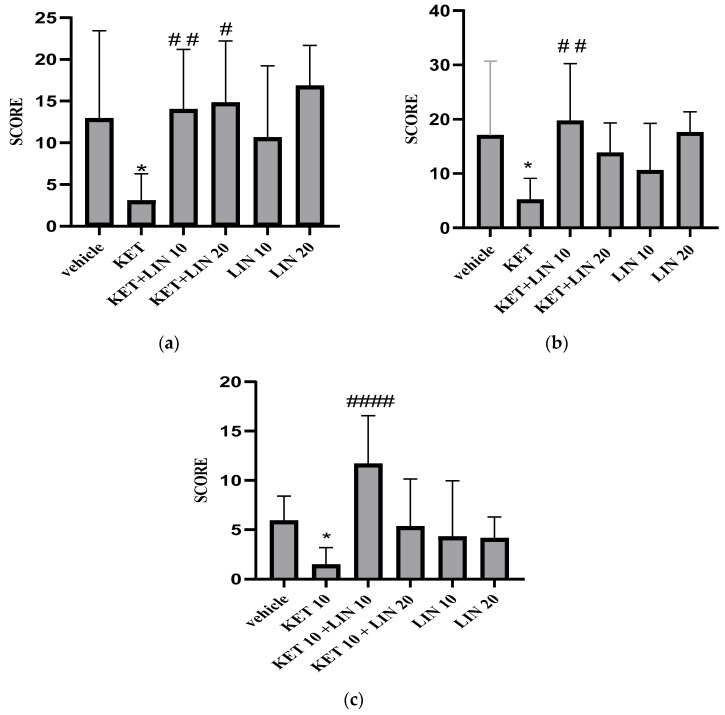
Effects of acute and chronic administration of linagliptin (LIN) (10 and 20 mg/kg, i.p.) on ketamine (KET) (10 mg/kg, i.p.)-induced memory impairments, in mice. In the acquisition model (**a**), linagliptin was administered 5 min after ketamine injection, and the passive avoidance test was conducted 45 min later. In the consolidation model (**b**), ketamine was administered first, followed by the behavioral test 50 min later; linagliptin was injected immediately after the test. In the chronic exposure model (**c**), linagliptin was administered once daily for 8 consecutive days. On the 8th day, ketamine was given 20 min after the final linagliptin dose. The initial passive avoidance test (TL1) was conducted 60 min after ketamine injection, and the retention test (TL2) was recorded 24 h later. Memory performance was assessed using the SCORE. Results are expressed as the mean SCORE ± SD. * *p* < 0.05 vs. vehicle group, # *p* < 0.05, ## *p* < 0.01, #### *p* < 0.0001 vs. ketamine group (N_B_ = 8–10, Tukey’s test).

Ketamine significantly impaired passive avoidance performance in the acquisition paradigm, as reflected by a reduced SCORE compared with the vehicle-treated group (3.08 ± 3.21, N = 10, *p* < 0.05). Administration of linagliptin at both 10 and 20 mg/kg significantly attenuated this ketamine-induced deficit, increasing the SCORE to 3.08 ± 3.21 (*p* < 0.01) and 14.80 ± 7.41 (*p* < 0.05), respectively, compared with the ketamine-treated group. Linagliptin administered alone did not significantly affect the SCORE relative to vehicle-treated animals (one-way ANOVA: F_5,56_ = 4.477, *p* = 0.0096; Figure 3a).

In the consolidation paradigm, ketamine also significantly reduced the SCORE compared with the vehicle group (5.18 ± 3.93, N = 10, *p* < 0.05). This effect was significantly attenuated by linagliptin at the dose of 10 mg/kg, which increased the SCORE to 19.72 ± 10.55 (*p* < 0.05 vs. ketamine). Administration of linagliptin alone did not significantly alter passive avoidance performance (one-way ANOVA: F_5,57_ = 6.610, *p* = 0.0011; Figure 3b).

Chronic ketamine administration likewise reduced the SCORE compared with vehicle-treated animals (1.43 ± 1.74, N = 10, *p* < 0.05). Repeated treatment with linagliptin at 10 mg/kg significantly attenuated this deficit, increasing the SCORE to 11.67 ± 4.88 (*p* < 0.0001 vs. ketamine), whereas the higher dose did not produce a statistically significant effect. Linagliptin administered alone had no significant effect on baseline passive avoidance performance (one-way ANOVA: F_5,58_ = 12.86, *p* < 0.0001; Figure 3c).

### 3.2. Effects of Acute and Chronic Linagliptin Administration on Diazepam-Induced Passive Avoidance Deficits in Mice

**Figure 4:**
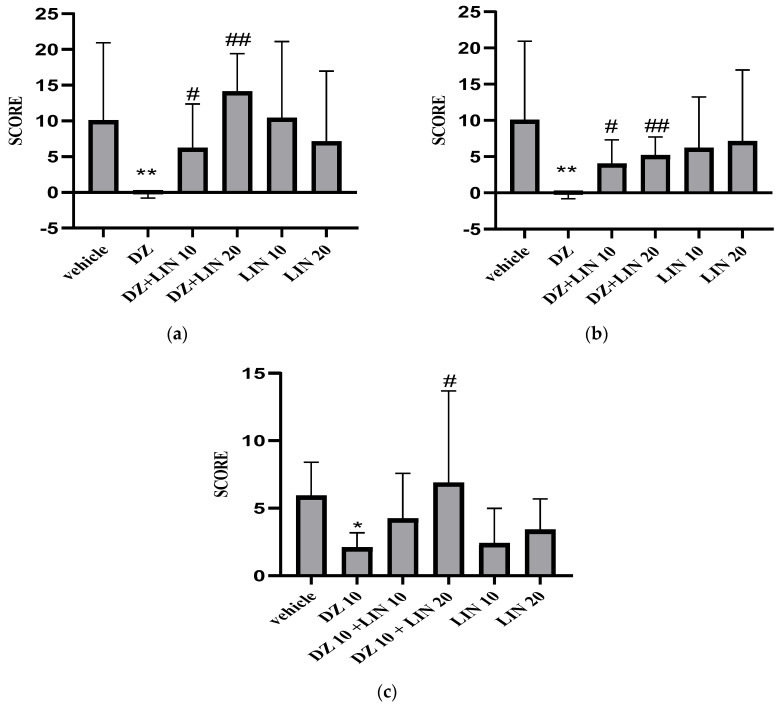
Effects of acute and chronic exposure to linagliptin (LIN) (10 and 20 mg/kg, i.p.) on diazepam (DZ) (10 mg/kg, i.p.)-induced memory impairments in mice. In the model of acquisition (**a**), diazepam was administered first, followed by linagliptin injection 20 min later; the passive avoidance test (TL1) was performed 45 min after the linagliptin injection. In the consolidation model (**b**), TL1 was recorded 65 min after diazepam administration, and linagliptin was injected immediately after the test; TL2 was recorded 24 h after the TL1 measurement. In the chronic exposure model (**c**), linagliptin was administered once daily for 8 consecutive days. On the 8th day, diazepam was given 20 min after the final linagliptin dose. The initial passive avoidance test (TL1) was conducted 60 min post-diazepam injection, and retention test (TL2) was recorded 24 h later. Memory performance was assessed using the SCORE. Results are expressed as the mean SCORE ± SD. * *p* < 0.05 vs. vehicle group, ** *p* < 0.01 vs. vehicle group, # *p* < 0.05, ## *p* < 0.01 vs. diazepam group (N_B_ = 8–10, Tukey’s test).

Diazepam significantly impaired passive avoidance performance in the acquisition paradigm, as reflected by a reduced SCORE compared with the vehicle-treated group (1.43 ± 1.74, N = 10, *p* < 0.01). Administration of linagliptin at both 10 and 20 mg/kg significantly attenuated this diazepam-induced deficit, increasing the SCORE to 11.67 ± 4.88 (*p* < 0.05) and 5.50 ± 4.85 (*p* < 0.01), respectively, compared with the diazepam-treated group. Linagliptin administered alone did not significantly affect the SCORE relative to vehicle-treated animals (one-way ANOVA: F_5,56_ = 2.415, *p* = 0.0453; Figure 4a).

In the consolidation paradigm, diazepam also significantly reduced the SCORE compared with the vehicle group (−0.18 ± 0.60, N = 9, *p* < 0.01). Both doses of linagliptin significantly attenuated this diazepam-induced deficit, increasing the SCORE to 4.02 ± 3.31 (*p* < 0.05) and 5.15 ± 2.59 (*p* < 0.01), respectively, compared with the diazepam-treated group. Administration of linagliptin alone did not significantly alter passive avoidance performance (one-way ANOVA: F_5,58_ = 3.967, *p* = 0.0296; Figure 4b).

Chronic diazepam administration likewise reduced the SCORE compared with vehicle-treated animals (2.08 ± 1.09, N = 10, *p* < 0.05). Repeated treatment with linagliptin at 20 mg/kg significantly attenuated this deficit, increasing the SCORE to 6.85 ± 6.84 (*p* < 0.05 vs. diazepam), whereas the lower dose did not produce a statistically significant effect. Linagliptin administered alone had no significant effect on baseline passive avoidance performance (one-way ANOVA: F_5,57_ = 4.139, *p* = 0.0119; Figure 4c).

### 3.3. Effects of Acute and Chronic Linagliptin Administration on BDNF Levels in the Prefrontal Cortex and Hippocampus

**Figure 5:**
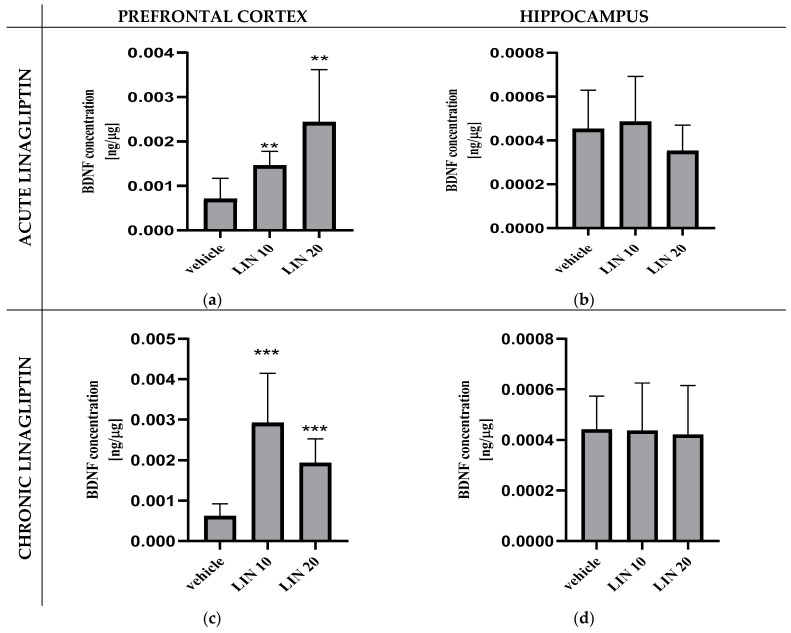
Effects of acute and chronic administration of linagliptin (LIN) (10 and 20 mg/kg, i.p.) on BDNF concentration in mouse. The prefrontal cortex (**a**,**c**) and the hippocampus (**b**,**d**) in mice. (**a**,**b**) Acute treatment: effect of a single linagliptin injection on BDNF levels in the (**a**) prefrontal cortex and (**b**) hippocampus. (**c**,**d**) Chronic treatment: effects of once-daily linagliptin administration (**a**,**b**) for consecutive days on BDNF levels in the (**c**) prefrontal cortex and (**d**) hippocampus. Data are presented as the mean ± SD. ** *p* < 0.01, *** *p* < 0.001 vs. vehicle group (N_I_ = 5–6, Mann–Whitney test).

Acute administration of linagliptin at doses of 10 and 20 mg/kg significantly increased BDNF levels in the prefrontal cortex compared with the vehicle group (0.0014 ± 0.0003, N = 6, *p* < 0.01 and 0.0024 ± 0.0012, N = 5, *p* < 0.01, respectively; Mann–Whitney U test; Figure 5a). In contrast, no significant changes in BDNF levels were observed in the hippocampus following acute linagliptin administration (Figure 5b).

Similarly, chronic administration of linagliptin for eight consecutive days significantly increased BDNF levels in the prefrontal cortex at both the 10 and 20 mg/kg doses (0.0029 ± 0.0012, N = 6, *p* < 0.001 and 0.0019 ± 0.0005, N = 6, *p* < 0.001, respectively; Mann–Whitney U test; Figure 5c). No statistically significant differences in hippocampal BDNF levels were detected following chronic treatment (Figure 5d).

## 4. Discussion

The present study demonstrates that the selective DPP-4 inhibitor linagliptin attenuates ketamine- and diazepam-induced deficits in passive avoidance performance in mice. In addition, both acute and chronic administration of linagliptin increased BDNF levels in the prefrontal cortex, whereas no significant changes were observed in the hippocampus. Although the present findings do not establish a causal relationship between these behavioral and biochemical effects, they provide preliminary evidence supporting the neuropharmacological activity of linagliptin in the central nervous system.

Linagliptin was administered at doses of 10 and 20 mg/kg, selected on the basis of preliminary studies performed in our Department showing that these doses did not affect blood glucose levels (unpublished observations). The same dose range was used in our previous studies investigating the neuropharmacological effects of linagliptin [28,33,34], in which no significant effects on locomotor activity, anxiety-like behavior, or depressive-like behavior were observed. Employing the same doses in the present study ensured methodological consistency and enabled direct comparison with our previous findings. Moreover, the selected doses are consistent with those commonly used in experimental studies evaluating the central effects of linagliptin in rodents [1,35,36].

To evaluate the behavioral effects of linagliptin, the passive avoidance test was employed. This paradigm is widely used to assess retention of an aversive experience and enables separate evaluation of the acquisition and consolidation phases of passive avoidance performance [37]. However, it is not intended to assess other cognitive domains, such as spatial or recognition memory. Albino mice exhibit a strong innate preference for dark environments [38], making this strain particularly suitable for passive avoidance studies. It should also be acknowledged that performance in this test may be influenced by factors other than learning, including anxiety, arousal, or locomotor activity. Nevertheless, in our previous studies, the same doses of linagliptin increased serotonergic neurotransmission [30] without affecting anxiety-like behavior in the elevated plus maze test [33]. Furthermore, linagliptin administered alone did not alter baseline passive avoidance performance in the present study. Collectively, these findings suggest that the effects of linagliptin observed in the passive avoidance test are unlikely to result from nonspecific alterations in anxiety-related behavior or locomotor activity.

To obtain a broader assessment of linagliptin activity, two pharmacological models producing passive avoidance deficits through distinct neurochemical mechanisms were employed. Ketamine, a non-competitive NMDA receptor antagonist, disrupts glutamatergic neurotransmission and impairs cognitive processing [39,40], whereas diazepam produces behavioral impairment through positive modulation of GABAA receptors, particularly α1- and α5-containing receptor subtypes [41,42,43,44]. Both compounds are widely used to induce deficits in passive avoidance performance in rodents [45,46,47,48]. In the present study, linagliptin attenuated ketamine-induced deficits following both acute and chronic administration, although the lower dose produced more consistent effects after repeated treatment. In the diazepam model, acute linagliptin administration improved passive avoidance performance in both the acquisition and consolidation paradigms, whereas chronic treatment was effective primarily at the higher dose. Taken together, these findings suggest that the behavioral effects of linagliptin are observed in pharmacological models involving both glutamatergic and GABAergic neurotransmission. However, the present experiments do not allow for conclusions to be drawn regarding the precise mechanisms underlying these effects.

As a selective DPP-4 inhibitor, linagliptin increases endogenous GLP-1 availability [3]. Beyond its well-established metabolic actions, accumulating evidence indicates that GLP-1 also exerts multiple effects within the central nervous system, including modulation of synaptic transmission, neuroinflammation, neuronal survival, and cognitive function [49,50,51,52,53,54,55]. Importantly, linagliptin has been shown to cross the blood–brain barrier [28], making central GLP-1 signaling a plausible mechanism underlying its neuropharmacological effects. Although several studies have suggested that DPP-4 inhibitors may improve cognitive function and exert neuroprotective actions [56], the underlying molecular mechanisms remain incompletely understood.

One pathway that has attracted considerable attention is the GLP-1/cAMP/CREB/BDNF signaling cascade [56]. Activation of GLP-1 receptors increases intracellular cAMP levels, leading to phosphorylation of CREB, a transcription factor that regulates BDNF synthesis [57]. Because BDNF plays a central role in neuronal survival, synaptic plasticity, and higher cognitive functions [58], we evaluated whether linagliptin administration alters BDNF levels in brain regions involved in cognitive processing. To our knowledge, this is the first study demonstrating that both acute and chronic administration of linagliptin are associated with increased BDNF levels in the prefrontal cortex.

Interestingly, no significant changes in BDNF levels were detected in the hippocampus. Although this finding may initially appear unexpected, region-specific regulation of BDNF has previously been reported [59,60,61,62]. The prefrontal cortex and hippocampus differ in their patterns of neuronal activity, intracellular signaling, and regulation of neurotrophin synthesis. Whereas cortical BDNF production appears to depend largely on cAMP-mediated signaling, hippocampal BDNF regulation is more strongly influenced by calcium-dependent mechanisms and other physiological factors [59,60]. Similar regional differences in BDNF responses have also been described following methamphetamine administration [61]. Furthermore, functional interactions between the hippocampus and prefrontal cortex are supported by GLP-1-sensitive neuronal circuits. Activation of GLP-1 receptors within the ventral hippocampus enhances phosphorylation of the NMDA receptor subunit NR2B in the medial prefrontal cortex, demonstrating functional communication between these structures [63]. Collectively, these observations suggest that the region-specific increase in prefrontal cortical BDNF observed in the present study is biologically plausible. However, the present data do not establish that increased BDNF levels directly mediate the behavioral effects of linagliptin.

The present findings should be interpreted in light of several limitations. First, passive avoidance represents a single behavioral paradigm and primarily assesses retention of an aversive experience. Although this test is widely used to investigate learning- and memory-related processes, additional behavioral paradigms, such as the novel object recognition test or spatial memory tasks, would provide a more comprehensive assessment of the cognitive effects of linagliptin. Furthermore, because ketamine and diazepam possess sedative, anesthetic, anxiolytic, and locomotor effects, some influence of these pharmacological actions on passive avoidance performance cannot be completely excluded. Nevertheless, the doses used in the present study have previously been validated in our laboratory and did not alter baseline behavioral performance. Moreover, linagliptin alone did not affect passive avoidance performance, supporting the view that the observed effects were not attributable to nonspecific behavioral alterations.

A second limitation concerns the biochemical analyses. BDNF levels were determined only in linagliptin-treated animals; therefore, the present results do not provide a comprehensive comparison across all experimental groups. In addition, BDNF quantification was normalized using a bicinchoninic acid (BCA) protein assay, which has recognized methodological limitations. Consequently, the biochemical findings should be interpreted with appropriate caution and confirmed in future studies using complementary analytical approaches.

Finally, although increased BDNF levels in the prefrontal cortex may represent one mechanism contributing to the neuropharmacological effects of linagliptin, the present experiments do not establish a causal relationship between BDNF regulation and the behavioral effects observed in the passive avoidance test. Other molecular pathways are also likely to participate in the central actions of linagliptin [64,65,66,67]. Therefore, the present findings should be regarded as preliminary evidence supporting the neurobehavioral effects of linagliptin and warrant further investigation using complementary behavioral paradigms and molecular approaches.

## 5. Conclusions

The present study provides preliminary evidence that linagliptin attenuates ketamine- and diazepam-induced deficits in passive avoidance performance in mice and is associated with increased BDNF levels in the prefrontal cortex. Although the underlying mechanisms remain to be established, these findings support further investigation of DPP-4 inhibitors as potential modulators of central nervous system function using complementary behavioral and molecular approaches.

## Data Availability

All data have been deposited to the Repository of Medical University of Lublin.

## Abbreviations

The following abbreviations are used in this manuscript:

BDNF	brain-derived neurotrophic factor
cAMP	cyclic adenosine monophosphate
CREB	cAMP response element-binding protein
DPP-4	dipeptidyl peptidase-4
ELISA	enzyme-linked immunosorbent assay
ERK	extracellular signal-regulated kinases
GABA	gamma-aminobutyric acid
GLP-1	glucagon-like peptide-1
MAPK	mitogen-activated protein kinase
NMDA	N-methyl-D-aspartate
VTA	ventral tegmental area
